# Brain MRI detection and classification: Harnessing convolutional neural networks and multi-level thresholding

**DOI:** 10.1371/journal.pone.0306492

**Published:** 2024-08-01

**Authors:** Rasool Reddy Kamireddy, Rajesh N. V. P. S. Kandala, Ravindra Dhuli, Srinivasu Polinati, Kamesh Sonti, Ryszard Tadeusiewicz, Paweł Pławiak

**Affiliations:** 1 Department of ECE, NRI Institute of Technology (Autonomous), Vijayawada, India; 2 School of Electronics Engineering (SENSE), VIT-AP University, Amaravati, Andhra Pradesh, India; 3 Department of ECE, VIEW, Vishakhapatnam, Andhra Pradesh, India; 4 Department of ECE, SVEC, Tadepalligudem, Andhra Pradesh, India; 5 Department of Biocybernetics and Biomedical Engineering, AGH University of Science and Technology, Krakow, Poland; 6 Department of Computer Science, Faculty of Computer Science and Telecommunications, Cracow University of Technology, Krakow, Poland; 7 Institute of Theoretical and Applied Informatics, Polish Academy of Sciences, Gliwice, Poland; National Textile University, PAKISTAN

## Abstract

Brain tumor detection in clinical applications is a complex and challenging task due to the intricate structures of the human brain. Magnetic Resonance (MR) imaging is widely preferred for this purpose because of its ability to provide detailed images of soft brain tissues, including brain tissue, cerebrospinal fluid, and blood vessels. However, accurately detecting brain tumors from MR images remains an open problem for researchers due to the variations in tumor characteristics such as intensity, texture, size, shape, and location. To address these issues, we propose a method that combines multi-level thresholding and Convolutional Neural Networks (CNN). Initially, we enhance the contrast of brain MR images using intensity transformations, which highlight the infected regions in the images. Then, we use the suggested CNN architecture to classify the enhanced MR images into normal and abnormal categories. Finally, we employ multi-level thresholding based on Tsallis entropy (TE) and differential evolution (DE) to detect tumor region(s) from the abnormal images. To refine the results, we apply morphological operations to minimize distortions caused by thresholding. The proposed method is evaluated using the widely used Harvard Medical School (HMS) dataset, and the results demonstrate promising performance with 99.5% classification accuracy and 92.84% dice similarity coefficient. Our approach outperforms existing state-of-the-art methods in brain tumor detection and automated disease diagnosis from MR images.

## 1. Introduction

Medical imaging plays a crucial role in clinical settings, providing valuable assistance to radiologists in patient analysis. Within medical imaging, the accurate detection and classification of brain tumors are particularly challenging and of utmost importance. In recent decades, research in medical imaging has flourished across various disciplines, including health monitoring, mathematics, computer science, engineering, and medicine.

Brain tumors are characterized by the abnormal and uncontrolled growth of cells within or around the brain. They are classified as non-malignant (benign) or malignant. Malignant tumors consist of cancerous cells that can spread to other parts of the body from their point of origin, whereas non-malignant tumors lack cancerous cells and do not metastasize. The World Health Organization (WHO) categorizes brain tumors into four grades: Grade I-IV. Grade I tumors are considered benign and typically pose a minimal threat. Grade II tumors are low-grade malignant tumors with a higher tendency to recur and progress to a higher grade. Grade III and IV tumors are classified as malignant and are generally more aggressive. Grade I tumors usually do not infiltrate nearby brain tissues, while grade II tumors may occasionally spread around brain tissues. In contrast, grade III and IV tumors are more likely to invade other brain tissues or even the spinal cord, making their treatment more challenging and posing significant risks to healthy brain tissues [1]. Therefore, early identification and accurate classification of such brain tumors are of paramount importance in medical imaging.

The advancement of medical imaging in brain tumor detection has been driven by the evolution of various scanning methods. Among these methods, Magnetic Resonance (MR) imaging has emerged as a widely utilized approach for examining brain images due to its exceptional ability to accurately differentiate soft tissues based on the characteristics of abnormal cells, including their location, shape, and size. Consequently, MR imaging plays a pivotal role in precise brain tumor detection [2]. Moreover, MR imaging offers the advantage of being non-invasive and provides multiple images with varying contrast visualizations of the same tissue, thereby furnishing radiologists with additional details during patient diagnosis.

Despite the benefits of MR imaging, clinical practitioners often face challenges as they require significant expertise and time to manually represent and classify brain tumors from MR images accurately. This manual process can introduce errors in the tumor detection procedure. To address these limitations, various research investigations have been conducted in recent years to identify and classify brain MR images more effectively, thereby minimizing these issues [3]. In the subsequent section, we present a review of some notable works in this field and summarize the key challenges encountered in brain tumor detection using medical imaging.

## 2. Literature review

Several studies have proposed different approaches for brain tumor detection using medical imaging. An unsupervised learning method for identifying brain tumors and segmenting tissues in MR images is presented in [4]. The approach uses a clustering technique that combines self-organizing maps (SOM) and fuzzy K-means (FKM) algorithms to extract features from the image. The identified features are then clustered into different classes based on the image intensity values. An improved automated brain tumor detection system was developed by Arunkumar et al. [5], using K-means clustering and artificial neural networks. (ANN). In order to implement an automated tumor detection model, Lu et al. [6] combined transfer learning with AlexNet. Nagapattinam et al. [7] proposed an automated CAD approach for brain tumor segmentation using genetic and adaptive neuro-fuzzy inference system (ANFIS) techniques.

Using hyper-column methods and attention modules, Toaçar et al. [8] designed the BrainMRNet architecture. A new deep-learning method that combines recursive feature elimination (RFE) and support vector machines (SVM) was introduced by Toaçar et al. [9]. By make use of support vector machines (SVMs) and multi-layer perceptron (MLP) an automatic method for brain tumors detection is presented in [10]. A two-stage classification framework for brain tumor identification using an SVM classifier is proposed in [11]. The first stage involves identifying tumor and non-tumor regions using an image processing technique called Image Difference of Smoothed Signals (IDSS). In the second stage, features extracted from the tumor regions are fed to the SVM classifier for classification into different tumor types.

An augmentation-based 2D convolutional neural network (CNN) system was proposed by Chanu et al. [12]. A new framework for segmenting and classifying brain tumors using deep convolutional neural networks (DCNN) was presented by Kuraparthi et al. [13]. Sethy et al. [14] implemented a deep feature fusion technique to distinguish brain MR images using VGG-16, principal component analysis (PCA) and SVM. In [15], a new method for segmenting abnormal parts in multimodal images by combining the kernel possibilistic C-means (KPCM) clustering algorithm, particle swarm optimization (PSO), and morphological reconstruction filters is proposed.

An automated tool for the early identification of brain tumors from MRI multimodal images are developed by combining the Cuckoo Search (CS) optimization algorithm with the K-nearest neighbor (KNN) classifier [16]. An improved multi-view fuzzy c-means clustering (IMV-FCM) algorithm is developed in [17]. The proposed algorithm aims to overcome the limitation of traditional methods by using multiple views of the image to capture the complex features and details of the brain tissues.

The CNN framework was used by Amin et al. [18] to identify and categories brain tumors. Arpit Kumar Sharma et al. [19] designed a technique based on the modified ResNet50 architecture and enhanced watershed (EWS) algorithm to distinguish between pathological and normal brain MR scans. In [20, 21], new deep-learning-based algorithms for predicting MR-based brain tumors are presented. In [22], a new approach for brain tumor segmentation from MRI images is presented. It combines the SOM and active contour model (ACM) techniques. The proposed approach, SOMACM, initializes the contour using SOM, which the ACM further refines. Wessam et al. [23] designed a classification framework for brain tumors based on variational auto-encoders and CNNs. Remzan et al. [24] developed a deep learning-based automatic tumor detection system.

Rahman et al. [25] proposed a parallel deep CNN (PDCNN) architecture to detect brain tumors in MRI images. Mohsen Ahmadi et al. [26] implemented a CNN and robust principal component analysis (RPCA) based approach for the identification of brain lesion in MR images. Sarah Zuhair Kurdi et al. [27] presented a meta-heuristic optimized CNN (MHO-CNN) architecture for the classification of brain MR images. Abdullah et al. [28] suggested a ML-based approach the identification of brain tumors using MR images. Ghada Saad et al. [29] developed a hybrid approach for the identification of brain abnormality from MR images using shape, and texture features. Jaber Alyami1et al. [30] proposed a novel approach for the localization of brain tumors from MR images using VGG, slap swam approach (SSA), and cubic-based SVM classifier. Table 1 illustrates the pros and cons of the existing studies.

**Table 1:** Pros and cons of the state-of-the-art methods.

Reference	Pros	Cons
Vishnuvarthanan et al. [4]	Efficient in terms of processing time and memory requirements.	Used limited data for validation.
Arunkumar N et al. [5]	Offers simplicity, and interpretability.	Limitations in handling complex shapes make it less ideal for brain tumor analysis.
Lu et al. [6]	Reducing the training time and computational resources.	Limited interpretability and fine-tuning challenges.
Nagarathinam et al. [7]	ANFIS to capture complex relationships within the data and improve classification of tumor regions.	ANFIS models can be complex to design and optimize, requiring careful selection of membership functions and fuzzy rules.
Toğaçar et al. [8]	Relatively preserve the valuable features due to the hypercolumn technique and minimizing the parameter redundancy	Potentially increasing the training time.
Toğaçar et al. [9]	RFE reduce the model complexity and easy to implement.	Greedy feature selection and depends upon the ranking criterion.
Kurmi Y et al. [10]	Mitigate the effects of noise and inhomogeneity. Handle more complex object boundaries	Smooth out details or shrink the segmented region, leading to information loss.
Polepaka S et al. [11]	Addresses low-contrast and non-Illumination issues.	The enhanced local binary patterns (E-LBP) might limit its ability to capture larger textural patterns.
Chanu MM et al. [12], and Remzan N et al. [24]	Eliminating the need for manual feature engineering, this can be time-consuming and error-prone.	Computationally expensive.
Kuraparthi S et al. [13]	Minimize the overfitting issues by data augmentation	Relying on pre-trained CNNs for feature extraction might limit control over the specific features used for classification.
Sethy PK et al. [14]	Fusing deep features extracted from different layers of the networks might capture complementary information, leading to better performance. Dimensionality reduction.	PCA assumes a linear relationship between the features. If the relationships are non-linear, it might not capture the most important information.
Sumathi R et al. [15]	KFCM allows for handling uncertainties in image segmentation, potentially leading to more robust results.	The effectiveness of the KFCM depends on choosing appropriate parameters, which can be challenging.
Sumathi R et al. [16]	Cuckoo Search (CS) is easy to implement and having a global search capability to finding the global optimum and avoiding getting stuck in local optima.	CS might converge slower than some other optimization algorithms like particle swarm optimization (PSO).
Hua L et al. [17]	IMV-FCM addresses the classic FCM algorithm’s sensitivity to noise by incorporating an adaptive learning mechanism.	Adding multiple views and an adaptive learning mechanism might increase the computational complexity of the algorithm compared to basic FCM.
Dehkordi AA et al. [18]	Nonlinear lévy chaotic moth flame optimizer (NLCMFO) improves the hyperparameter tuning ability to find the optimal parameters.	NLCMFO likely involves several parameters for both Lévy flights and chaotic search that need to be tuned for optimal performance. This can be challenging and require problem-specific adjustments.
Sharma AK et al. [19]	Enhanced watershed algorithm significantly handling the over segmentation problems.	Leads to under segmentation.
Sharma S et al. [20], and Alsaif H et al. [21]	VGG relatively extracts rich and hierarchical features from images.	High computational cost and prone to overfitting.
Sandhya G [22]	SOMACM can be effective for segmenting complex objects with irregular shapes or weak boundaries in images.	Combining SOMs and ACMs adds complexity to the segmentation process compared to using a basic ACM. This can lead to higher computational cost and potentially make it less efficient.
Salama WM [23]	Convolutional variational autoencoders (CVAE) allows them to capture spatial relationships in images, making them suitable for learning complex image distributions with intricate details.	Training CVAEs can be more complex compared to simpler autoencoder architectures.
Rahman T et al. [25]	Faster training and scalability.	Complexity of implementation
Ahmadi M et al. [26]	Robust PCA (RPCA) effectively handling outliers and noise in the data.	RPCA can be computationally more expensive compared to standard PCA.
Kurdi SZ et al. [27]	MHO-CNN improves the hyperparameter tuning and handles the high dimensionality of the hyperparameter space in complex CNN architectures.	No guarantee of global optimum.
Saad G et al. [29]	Computationally efficient.	Sensitivity to noise.
Alyami J et al. [30]	Simple and easy to implement.	Slow convergence and limited theoretical understanding.

### 2.1. Research gaps

From the above-mentioned conventional brain tumor identification and classification approaches, we identify the following research gaps:

1. While some approaches have employed traditional local texture feature extraction methods like local binary patterns (LBPs) [11], these techniques are sensitive to illumination variations, random noise, and rotations. As a result, their robustness and accuracy in detecting brain tumors may be compromised.
2. Certain studies [7, 10] have utilized statistical texture features to differentiate between normal and abnormal brain MR images. However, they do not account for the spatial correlation between adjacent pixels, potentially limiting their ability to capture important spatial information relevant to accurate tumor detection.
3. Many existing approaches neglect data augmentation [8, 9, 19, 29], leading to lower classification accuracy. Machine learning and deep learning models often rely on a sufficient amount of data for optimal performance. The lack of data augmentation can hinder the model’s ability to generalize well to new and unseen cases.
4. Several studies have resorted to pre-trained CNN models like ResNet-50, VGG, and AlexNet [6, 9, 14, 20, 21, 23, 24]. However, employing these models requires a large number of parameters, resulting in increased computational complexity and potentially limiting their practicality in real-time applications.

To address these challenges and enhance brain tumor detection accuracy while mitigating overfitting and reducing computational complexity, we propose a novel framework. This distinctive approach incorporates advanced texture feature extraction methods, considering spatial correlations, and integrates data augmentation techniques to boost classification performance. Additionally, our framework introduces different strategies to optimize and streamline the training of deep learning models, ensuring efficient and accurate tumor detection in medical imaging. By bridging these research gaps, our approach aims to significantly improve brain tumor diagnosis and classification in clinical settings.

The article is structured as follows: Section 3 presents the proposed techniques. Section 4, the outcomes of the segmentation and classification of the proposed and state-of-the-art frameworks are compared and analyzed. Section 5 highlights the major findings of the proposed framework. Finally, Section 6 presents the conclusion of the study.

## 3. Proposed methodology

**Fig 1:**
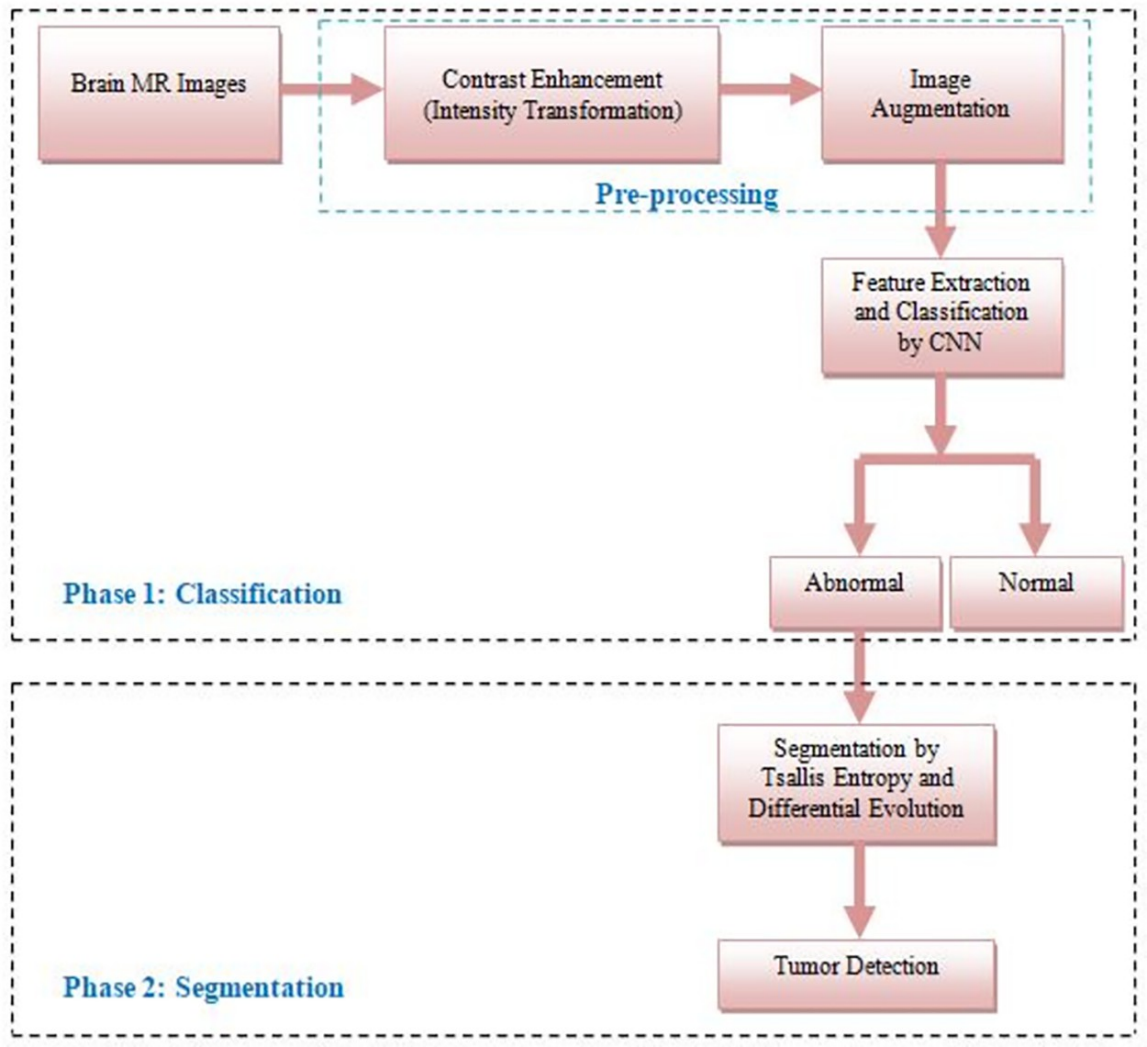
The proposed brain tumor segmentation and classification approach.

Detecting and categorizing brain tumors through MRI images can be challenging because of the differences in the characteristics of tumors. In this study, we introduce an innovative approach that utilizes CNN and multi-level thresholding to overcome this issue. Our proposed system comprises two components: classification and segmentation, as shown in Fig 1. The subsequent sections provide a comprehensive overview of these phases.

### 3.1. Classification phase

The brain MR image classification process consists of two stages. In the first stage, an intensity transformation operator is used to adjust the contrast of the images. In the second stage, image data augmentation techniques are applied to enhance the model performance by reducing overfitting.

#### 3.1.1. Database

To evaluate the performance of the proposed model, we collected 264 T2-weighted brain MR images with 256×256 resolution from a publicly available database, namely Harvard medical school [31], which includes 194 abnormal and 70 normal subjects. However, this dataset may not be sufficient to build an efficient model. Therefore, further, we employ data augmentation described in section 3.1.3.

#### 3.1.2. Contrast enhancement

MRI images of the brain often suffer from unwanted information or artifacts, which can occur during the scanning process. In some cases, artifacts present in brain MR images can make it challenging for radiologists to accurately identify or extract the region of interest, especially when abnormalities are present. To address this issue, we utilized an intensity transformation method to increase the contrast of the images and improve their overall quality. For this purpose, we used a MATLAB built-in function, imadjust [32]. This function adjusts the contrast of an image by stretching its intensity values to cover the full dynamic range. This function maps the input image’s intensities to new values spanning a specified range of pixel values in the output image. Here, we limit the low and high pixel values between 0.01 and 0.99. By this, we can improve the contrast of brain MR images. After that, we applied data augmentation to improve the model classification accuracy.

#### 3.1.3 Data augmentation

The CNN models heavily depend on the size and diversification of data to minimize overfitting problems. However, many application domains lack diversified and massive data, especially medical image analysis. Therefore, to enhance the performance of our proposed framework and minimize overfitting, we utilized image data augmentation techniques. These techniques involved applying geometric transformation operators, including scaling, translation, reflection, shearing, and rotation, to create a more diverse set of input data with the help of configurations mentioned in the work [33]. This process induced 2376 brain MR images from the dataset mentioned in section 3.1.1. The augmented data raised the original data to 2376 MR images, 1746 abnormal and 630 healthy. After that, we employed the suggested CNN framework on these augmented images to detect abnormal MR images.

#### 3.1.4. The proposed CNN architecture

CNN is a typical neural network model that proved efficient in image classification and recognition. Usually, they include convolutional layers (that apply convolution operation on the input data), activation layers (introduce non-linearity into the data), batch normalization or batch norm (enhance the network stability), pooling layers (that down-sample the spatial dimensions of the data), fully connected layers (that use standard multi-layer perceptron architecture) and softmax (estimate the class probabilities) [33]. CNNs have some critical advantages over traditional neural networks [34], such as:

- **Parameter Sharing:** CNNs use shared weights in the convolutional layers, which reduce the number of parameters to learn, leading to reduced overfitting and increased efficiency.
- **Spatial Invariance:** CNNs have a property called spatial invariance, meaning that the network is translation invariant and can detect the same feature anywhere in the input.
- **Downsampling:** CNNs have pooling layers that down-sample the spatial dimensions of the data, reducing the number of parameters to learn and the computational cost, making the network less prone to overfitting.

CNNs have proven to be effective in image classification and computer vision tasks due to their properties stated above. Prior studies have developed conventional deep learning models such as ResNet-50, VGG, and AlexNet to detect abnormalities in brain MR images [6, 8, 12, 13, 15, 24, 25]. However, these models require many parameters, leading to increased computational complexity. To alleviate this issue, we suggested a lightweight CNN architecture that reduces the number of trainable and non-trainable parameters, thereby reducing training time without compromising classification performance.

**Fig 2:**
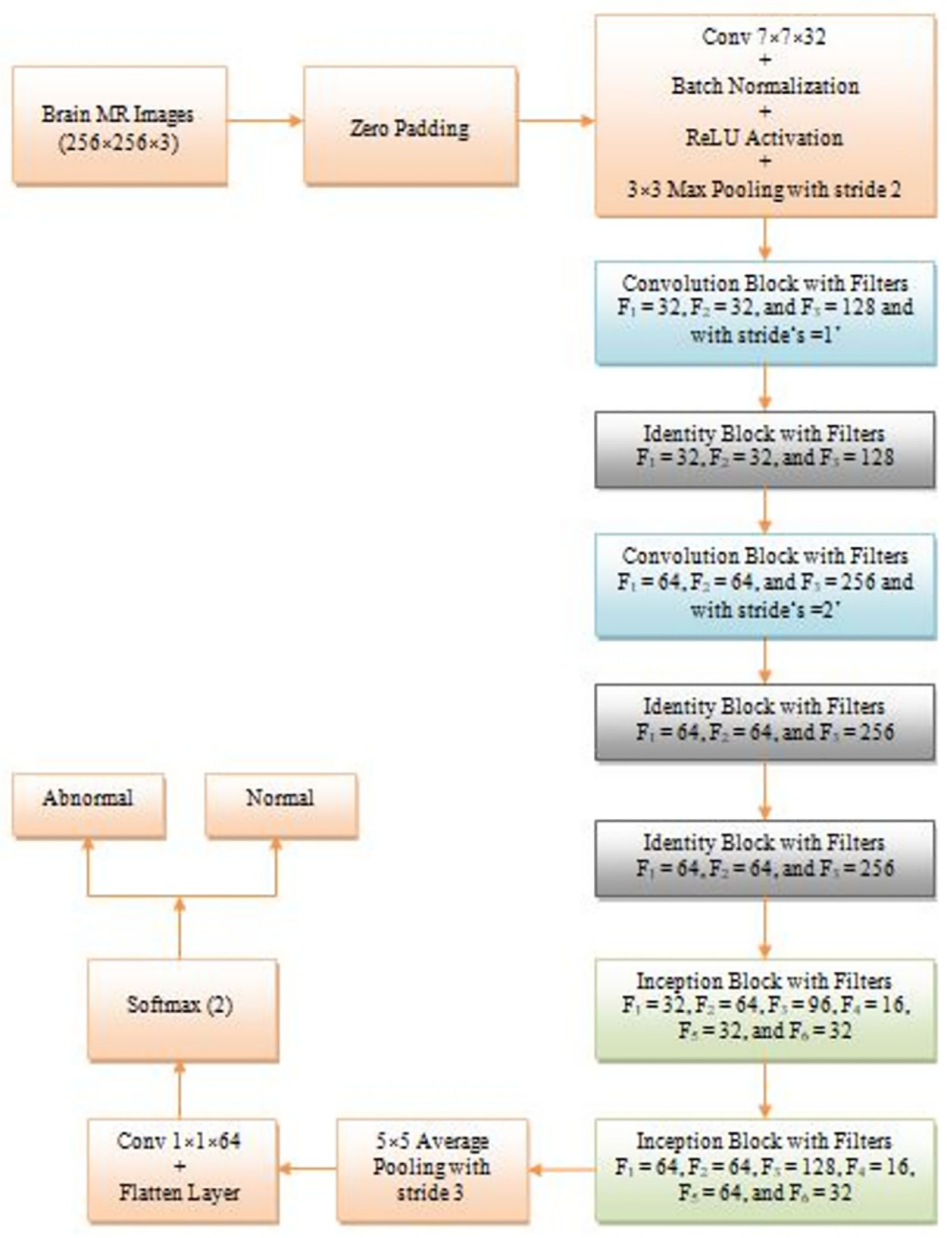
The suggested CNN framework.

**Fig 3:**
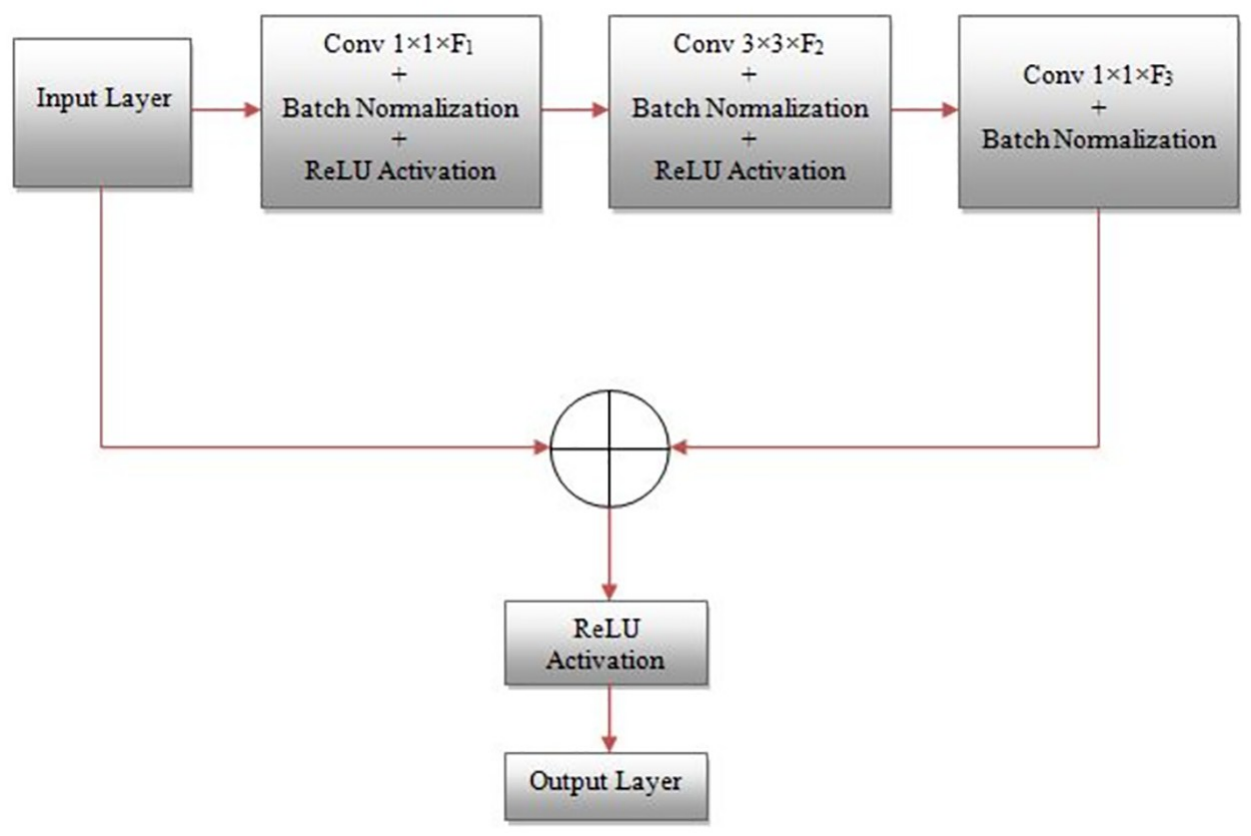
Block diagram of the identity block.

**Fig 4:**
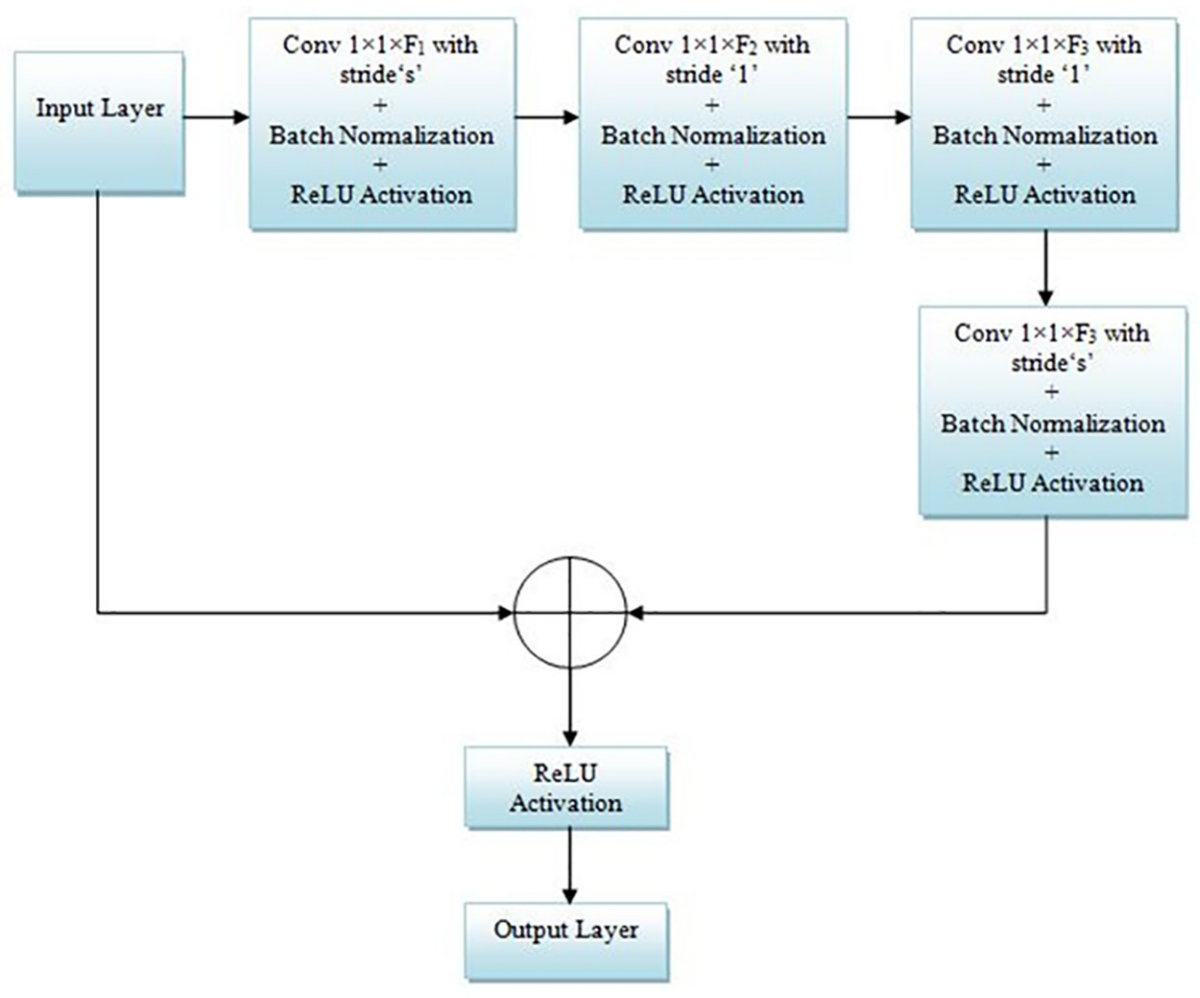
Block diagram of the convolution block.

**Fig 5:**
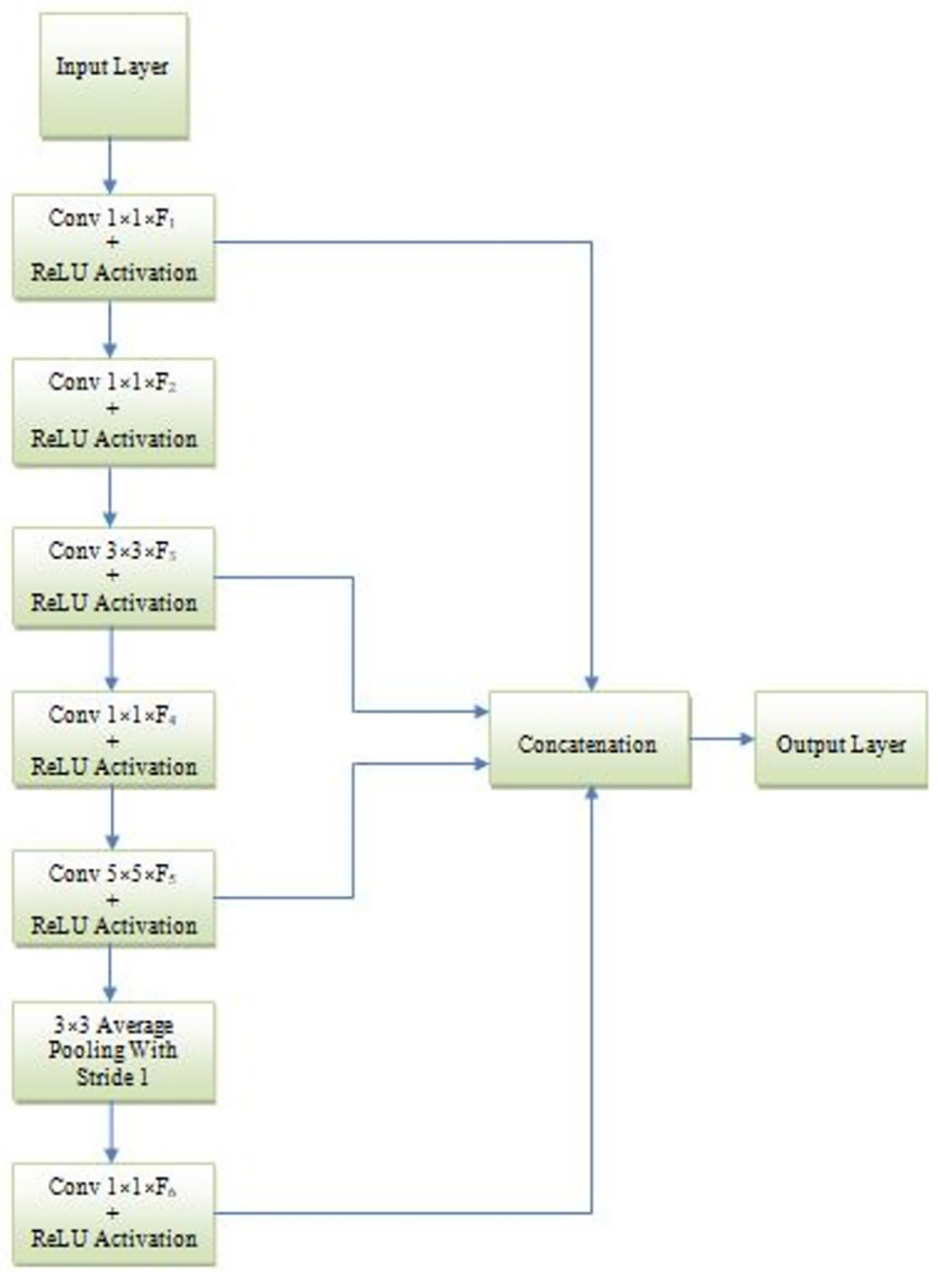
Block diagram of the inception block.

Fig 2 represents the architecture of our lightweight CNN framework. It consists of three fundamental blocks, namely, Convolutional, Identity, and Inception, shown in Figs 3–5, in addition to zero padding, average pooling, and a softmax layer. Here, initially, zero padding was applied to preserve the characteristics of the image at the edges and control the dimensions of the output feature map. Then, a sequence of layers was employed to attain significant edge features such as a 7×7 convolution layer with 32 filters, batch norm, ReLU activation, and 3×3 max pooling with stride 2. After that, we used a series of convolutional, identity, and inception blocks with specified filters, namely, F1, F2, F3, F4, F5, and F6. The significance of these blocks is described in the subsequent sections.

*A*. *Identity block*. Identity networks, also known as identity-based networks or identity mappings, are deep neural network architectures that use identity maps to improve learning. The primary goal of identity networks is to allow for deeper architectures that are easier to train and converge than traditional deep neural networks. It is achieved by adding identity maps as skip connections that bypass one or more layers and map inputs directly to outputs. The identity maps provide a direct path for the gradients to flow during backpropagation, preventing the vanishing or exploding gradients problem and enabling the network to learn more effectively. The concept of identity networks was introduced in the ResNet (Residual Network) architecture, which demonstrated state-of-the-art performance in computer vision tasks. Since then, identity-based architectures have become popular and widely used in various fields, including computer vision, speech recognition, and natural language processing.

The suggested identity network used in implementing our model is shown in Fig 3. It mainly consists of three convolution modules (two 1×1 and one 3×3) with filter sizes F1, F2, and F3, respectively. In addition, each convolution module is preceded by batch norm and ReLU activation layer. We employed three identity networks in the proposed model with F1 = 32, 64; F2 = 32, 64; F3 = 64, 256.

*B*. *Convolution block*. The main motive of the proposed CNN frameworks is to achieve high accuracy with low-computational cost. To meet this criterion, we introduced the ‘Convolution’ module into the presented architecture, represented in Fig 4. The suggested convolution module has four 1×1 convolution blocks with filters F1, F2, and F3, and followed by a batch norm and ReLU activation. The 1×1 convolution is primarily utilized in [35] for cross-channel pooling, but later it is employed in implementing modern architectures such as GoogleNet, ResNet, SqueezeNet, and Inception-ResNet since

1. It reduces the number of feature maps.
2. Reduce the computational cost by minimizing the parameter map.
3. Introduce the non-linearity into the network.
4. Create smaller architectures that retain a higher degree of accuracy.

In the suggested CNN architecture, we used two convolution blocks with F1 = 32, 64; F2 = 32, 64; and F3 = 64, 128 with stride s = 1 and 2.

The proposed CNN model includes a 5×5 average pooling with stride 3, a 1×1 convolutional layer with 64 filters, and a softmax layer. These layers were incorporated before the outcomes were passed to the segmentation phase, which identifies the affected region of pathological brain MR images.

*C*. *Inception block*. Inception is a type of deep neural network architecture introduced in 2014 by Google researchers. The name “Inception” refers to the architecture’s ability to examine multiple scales or “aspects” of the input data simultaneously, in a parallel manner. It is achieved using multiple branches with different kernel sizes in the convolutional layers, allowing the network to capture features at different scales. The multiple branches then concatenate their output activations to form a combined feature representation, which is then passed on to the next layer. This architecture helps reduce overfitting, improve accuracy, and allow for more efficient use of computation resources. The Inception architecture has gained widespread popularity in the field of computer vision, particularly for image classification tasks. Its impact on the development of deep learning models for a variety of applications has been significant.

The proposed inception network utilized in the implementation of our model is shown in Fig 5. It mainly consists of six convolution modules (four 1×1, one 3×3, and one 5×5) with filter sizes F1, F2, F3, F4, F5, and F6 and each convolution module is followed by a ReLU activation layer. In our model, we applied two inception network architectures with F1 = 32, 64; F2 = 64, 64; F3 = 96, 128; F4 = 16, 16; F5 = 32, 64; and F6 = 32, 32. The entire flow of the proposed classification model is illustrated in the Algorithm 1.

*D*. *Inception block*. Inception is a type of deep neural network architecture introduced in 2014 by Google researchers. The name “Inception” refers to the architecture’s ability to examine multiple scales or “aspects” of the input data simultaneously, in a parallel manner. It is achieved using multiple branches with different kernel sizes in the convolutional layers, allowing the network to capture features at different scales. The multiple branches then concatenate their output activations to form a combined feature representation, which is then passed on to the next layer. This architecture helps reduce overfitting, improve accuracy, and allow for more efficient use of computation resources. The Inception architecture has gained widespread popularity in the field of computer vision, particularly for image classification tasks. Its impact on the development of deep learning models for a variety of applications has been significant.

The proposed inception network utilized in the implementation of our model is shown in Fig 5. It mainly consists of six convolution modules (four 1×1, one 3×3, and one 5×5) with filter sizes F1, F2, F3, F4, F5, and F6 and each convolution module is followed by a ReLU activation layer. In our model, we applied two inception network architectures with F1 = 32, 64; F2 = 64, 64; F3 = 96, 128; F4 = 16, 16; F5 = 32, 64; and F6 = 32, 32. The entire flow of the proposed classification model is illustrated in the Algorithm 1.

**Algorithm 1:** MRI Classification and Tumor Segmentation

**Input:** Original MR-T2 images: original images (194 abnormal, 70 normal)

**Output:** Classified images: classified_images (normal/abnormal labels)

**Steps:**

 1. **Pre-processing:** For each image in original_images:

  • Apply “imadjust” function to enhance contrast and normalize intensity range to [0.01, 0.99].

  • Store the pre-processed images in preprocessed_images.

 2. **Data Augmentation:**

  • Apply data augmentation techniques (e.g., flipping, scaling, and rotation) to preprocessed_images to create a larger dataset augmented_images.

  • Ensure augmented_images maintain a balanced class distribution (normal/abnormal).

 3. **CNN Model:** Define a lightweight CNN architecture with:

  • Convolutional blocks for feature extraction.

  • Identity blocks for gradient flow improvement.

  • Inception blocks for efficient feature reuse.

  • Zero padding to maintain image size during convolution.

  • Average pooling for dimensionality reduction.

  • Softmax layer for final classification (normal/abnormal).

  • Train the CNN model on augmented_images with appropriate loss function and optimizer.

 4. **Classification:**

  • Use the trained CNN model to classify each image in preprocessed_images.

  • Store the predicted labels (normal/abnormal) in classified_images.

### 3.2. Segmentation

Segmentation is essential in various image-processing applications, such as medical imaging, content-based image retrieval, and computer vision. Medical imaging is imperative to identify the region of interest (ROI) in patients with brain-related diseases. In this study, our approach aims to differentiate the normal and abnormal regions of brain MR images by thresholding and morphological operations.

We first pre-process the brain MR images and estimate the global thresholding value using Tsallis entropy-based multi-level thresholding and differential evolution (DE) to accomplish this. This allows us to differentiate the affected and unaffected areas of the image using the estimated thresholding value. However, this process can lead to imperfections in the obtained threshold image. To address this issue, we perform post-processing using morphological operations [32, 36]. This method ensures the accuracy of the segmentation results and allows us to identify the ROI for further analysis.

#### 3.2.1. Multi-level Tsallis entropy

In the context of image processing, entropy relatively stores the precise variations contained in the image. Let us assume that *J* be an input image with the dimensions of *U* × *V* and the corresponding normalized histogram of the image *J* is denoted by *H* = (*h*_1_, *h*_2_, *h*_3_…, *h*_*m*_), where $h_{m}=\frac{r_{m}}{\left(U\times V\right)},\;0\leq m<L$; *r*_*m*_ be the number of occurrences of gray-level *m* and *L* is the total number of gray-levels of *J* (usually 255). Now, partition the image into *m*+1 classes by *m* thresholds (*T*) and then estimate the Tsallis entropy [37] for each partition using Eq (1):

$$H_{1}\left(T\right)=\frac{1-\sum_{k=0}^{T_{1}}{\left(\frac{p_{k}}{P_{1}}\right)}^{\gamma }}{\gamma -1};H_{2}\left(T\right)=\frac{1-\sum_{k=T_{1}+1}^{T_{2}}{\left(\frac{p_{k}}{P_{2}}\right)}^{\gamma }}{\gamma -1};H_{m}\left(T\right)=\frac{1-\sum_{k=T_{m}+1}^{L-1}{\left(\frac{p_{k}}{P_{m}}\right)}^{\gamma }}{\gamma -1}$$ (1)

where γ represents an entropy index and it is always a real value.


$$P_{1}\left(T\right)=\sum_{k=0}^{T_{1}}h_{k},P_{2}\left(T\right)=\sum_{k=T_{1}+1}^{T_{2}}h_{k},\ldots ,P_{m}\left(T\right)=\sum_{k=T_{m}+1}^{L-1}h_{k}.$$ (2)

To attain the significant threshold value *T*_*opt*_, the total Tsallis entropy function must be maximized as follows:

$$T_{opt}=\text{arg}\;\text{max}\left(\left[H_{1}\left(T\right)+H_{2}\left(T\right)+H_{3}\left(T\right)+\ldots +H_{m+1}\left(T\right)\right]\right)$$ (3)

**Fig 6:**
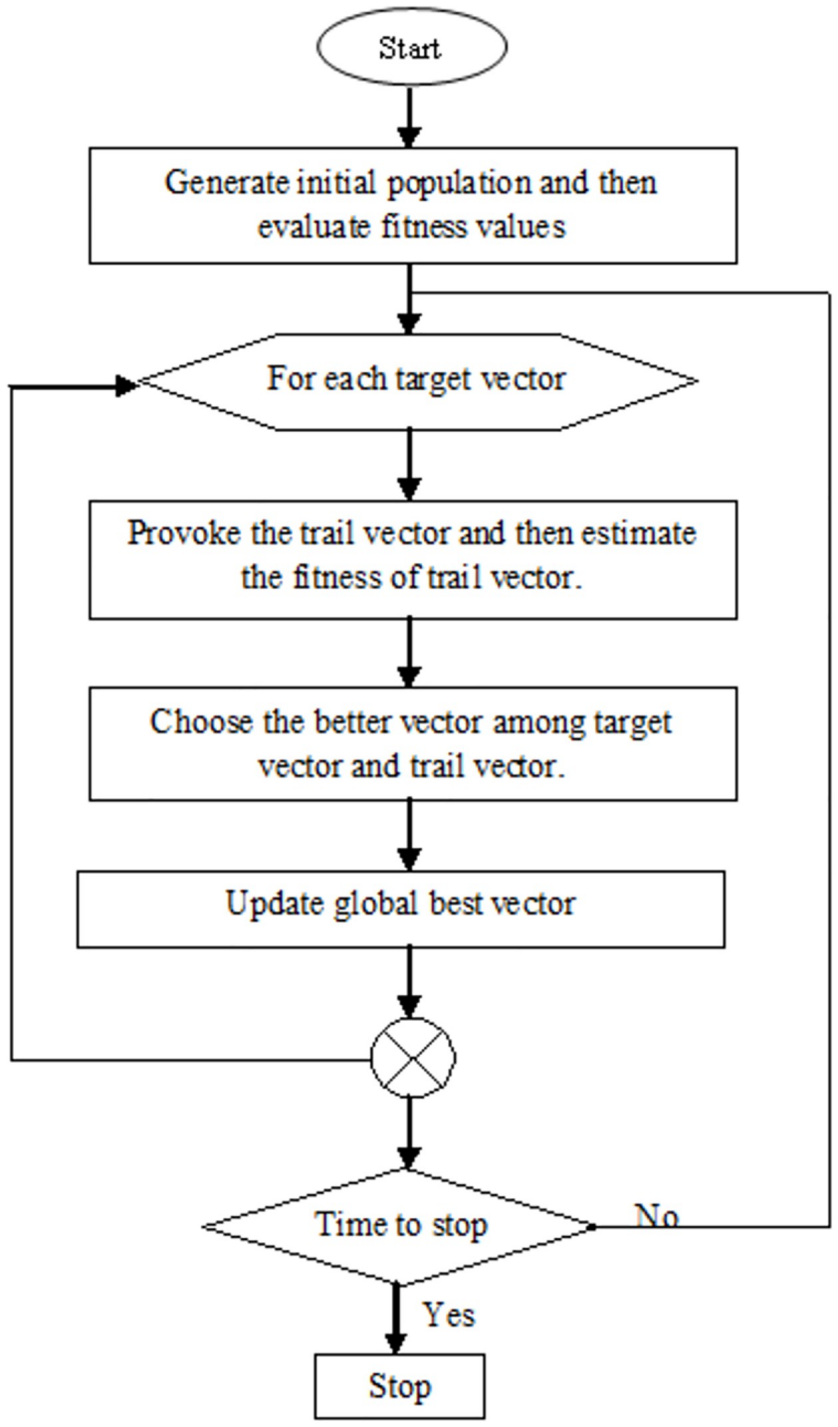
Flow diagram of the differential evolution.

The above task (Eq (3)) is achieved by a population-based meta-heuristic global optimization approach known as Differential Evolution [38]. It is a simple and efficient evolutionary approach than other existing evolution frameworks like a genetic algorithm (GA) [39], and particle swarm optimization (PSO) [40]. Here, it is maximized the entropy function by iteratively enhancing a candidate solution subject to an evolutionary procedure. The whole process of DE is illustrated in Fig 6. After performing the thresholding, for an effective outcome, furthermore, we employed post-processing using mathematical morphology concepts.

#### 3.2.2. Post-processing

Post-processing is an essential step in the segmentation process to remove imperfections that occur in the threshold image. It involves morphological image operations such as erosion and dilation, taking into account the shape and boundary area of the tumor. By performing these operations on the threshold image, we can improve the accuracy of tumor detection in brain MR images. A disk-shaped template with a radius of ten is typically used for this purpose. Algorithm 2 outlines the proposed segmentation algorithm.

**Algorithm 2:** The proposed segmentation approach

 1. Read the enhanced brain MR image, *J*.

 2. Perform multi-level Tsallis entropy using the process outlined in section 3.2.1.

 3. Employ DE approach to optimize the entropy function. To achieve this, here, we consider the following parameters:

  Number of thresholds = 6,

  Optimization parameters (*D*) = 12,

  Population size (*NP*) = 10 × D,

  Weighting factor (*F*) = 0.5,

  Cross-over probability (*CR*) = 0.9,

 4. To obtain an appropriate thresholding value (*T*_*a*_), we consider the mean of the first three largest thresholding values.

 5. Obtain the segmented image by applying the binarization process using the threshold attained in step 4.

 6. For significant brain tumor segmentation, finally, we employed post-processing which is described in section 3.2.2.

### 3.3. Evaluation measures

The presented framework is assessed through the following metrics [41]:

$$\text{Accuracy}=\frac{TP+TN}{TP+TN+FP+FN}$$ (4)

$$\text{True}\;\text{Positive}\;\text{Rate}\;(\text{TPR})=\frac{TP}{TP+FN}$$ (5)

$$\text{True}\;\text{Negative}\;\text{Rate}\;\;(\text{TNR})=\frac{TN}{TN+FP}$$ (6)

$$\text{Positive}\;\text{Predictive}\;\text{Value}\;(\text{PPV})=\frac{TP}{TP+FP}$$ (7)

$$\text{F-Score}=2\left(\frac{PPV\times TPR}{PPV+TPR}\right)$$ (8)

$$\text{Dice}\;\text{Similarity}\;\text{Coefficient}\;(\text{DSC})=\frac{2\times \left|T\cap T_{G}\right|}{\left|T\right|+\left|T_{G}\right|}$$ (9)

$$\text{Area}\;\text{Under}\;\text{Curve}\;(\text{AUC})=\left(\frac{TPR+TNR}{2}\right)$$ (10)

where, *T* = Segmented image; *T*_*G*_ = Ground truth; *TP* = True Positive; *TN* = True Negative; *FP* = False Positive, and *FN* = False Negative.

## 4. Experimental results

The simulation results of the suggested approach are presented in the section. To test the reliability of our framework, we conducted extensive simulations based on K-fold cross-validation (K-FCV). Generally, K-FCV is an easy and effective technique for reducing overfitting compared to other validation strategies [42]. However, choosing of K is a crucial part of the validation process. A model with a low variance and a high bias will result from a smaller K-fold sample size. In a similar vein, when the K-fold parameter is significantly increased, the model becomes overfit. Hence, we chose the number 5 for K because it seemed like a good compromise between reliability and randomness. For ease of comprehension, the presented model simulation results are subdivided into two sections. Here, the first module identifies the abnormality of brain MR images. The second module deals with the segmentation of pathological brain MR images.

### 4.1. Identification of brain abnormality

We used the suggested CNN framework to predict brain MR image abnormality. Our model was trained on contrasted enhanced augmented images, allowing it to automatically detect relevant edge details through hidden layers and backpropagation learning. To optimize the training process, we utilized a batch size of 64 and trained for 30 epochs. We also experimented with various optimizers to minimize loss, including SGDM [43], AdaMax [44], Adam [44], Adagrad [45], Adadelta [46], RMSProp [47], and Nadam [48], and the specific hyperparameters for each are listed in Table 2.

**Table 2:** Various optimization algorithms’ parameters considered in this work.

Optimization	Parameters
SGDM	*α* = 0.001, momentum = 0.9
Adam	*α* = 0.001, *β*_1_ = 0.9, *β*_2_ = 0.999, and *ε* = 1e-07
Adamax	*α* = 0.001, *β*_1_ = 0.9, *β*_2_ = 0.999, and *ε* = 1e-07
Adagrad	*α* = 0.001 and *ε* = 1e-07
Adadelta	*α* = 0.001, *ε* = 1e-07 and rho = 0.95
RMSprop	*α* = 0.001, *ε* = 1e-07 and rho = 0.9
Nadam	*α* = 0.001, *β*_1_ = 0.9, *β*_2_ = 0.999, and *ε* = 1e-07

**Note:** *α***:** learning rate; *β*_1_, *β*_2_: ‘rho’ and decay factors; *ε*:constant (considered a smaller value for numerical stability)

Tables 3–9 display the performance of our strategy on various optimizers. It is evident from these tables that Adadelta underperforms compared to other optimizers, particularly in identifying healthy brain MR images due to the drastic decrease in learning rate in the later stages of training. We found that Adagrad and RMSProp outperformed Adadelta by about 95%, but this level of accuracy is not acceptable for clinical diagnosis. On the other hand, SGDM, Adam, AdaMax, and Nadam produced relatively high accuracy, averaging at around 99%. However, among these optimizers, Adam and Nadam significantly improved the proposed technique’s performance by effectively minimizing the loss function. They achieved 99.66% TPR, 99.2% TNR, 99.71% PPV, 99.52% F-Score, 99.42% AUC, and 99.5% accuracy on the proposed CNN architecture. Primarily it is because they slow down when converging to the local minima and minimize the high variance.

**Table 3:** Performance measures of the proposed CNN architecture: SGDM optimization.

5-FCV (Fold wise)	Evaluation Measures (%)					
	TPR	TNR	PPV	F-Score	AUC	Accuracy
1	99.7	98.60	99.40	99.55	99.15	99.37
2	100	97.62	99.14	99.57	98.81	99.37
3	100	98.55	99.41	99.7	99.27	99.58
4	98.91	98.11	99.45	99.18	98.51	98.74
5	99.72	94.87	98.34	99.02	97.3	98.52
Mean ± SD	99.67±0.2	97.55±0.7	99.15±0.21	99.40±0.13	98.60±0.35	99.12±0.2

**Table 4:** Performance measures of the proposed CNN architecture: Adam optimization.

5-FCV (Fold wise)	Evaluation Measures (%)					
	TPR	TNR	PPV	F-Score	AUC	Accuracy
1	100	100	100	100	100	100
2	99.7	99.31	99.7	99.7	99.5	99.37
3	99.15	97.52	99.15	98.33	98.33	98.74
4	99.73	100	100	99.86	99.86	99.8
5	99.72	99.16	99.72	99.72	99.44	99.58
Mean ± SD	**99.66±0.14**	**99.19±0.45**	**99.71±0.15**	**99.52±0.3**	**99.42±0.29**	**99.5±0.21**

**Table 5:** Performance measures of the proposed CNN architecture: Adamax optimization.

5-FCV (Fold wise)	Evaluation Measures (%)					
	TPR	TNR	PPV	F-Score	AUC	Accuracy
1	100	94.91	98.35	99.17	97.45	98.74
2	100	99.21	99.71	99.85	99.61	99.8
3	99.72	94.78	98.35	99.21	97.25	98.52
4	99.7	98.58	99.4	99.55	99.14	99.37
5	100	95.52	98.3	99.14	97.76	98.74
Mean ± SD	99.88±0.07	96.6±0.94	98.82±0.3	99.38±0.14	98.24±0.47	99.03±0.23

**Table 6:** Performance measures of the proposed CNN architecture: Adadelta optimization.

5-FCV (Fold wise)	Evaluation Measures (%)					
	TPR	TNR	PPV	F-Score	AUC	Accuracy
1	96.83	72.86	90.56	93.60	84.84	90.33
2	95.96	71.87	90.24	93.01	83.91	89.47
3	95.78	78.99	93.16	94.45	87.38	91.58
4	95.08	73.64	90.63	92.80	84.36	89.26
5	94.85	70.4	89.97	92.34	82.62	88.42
Mean ± SD	95.7±0.35	73.55±1.46	90.91±0.57	93.24±0.36	84.62±0.78	89.81±0.53

**Table 7:** Performance measures of the proposed CNN architecture: Adagrad optimization.

5-FCV (Fold wise)	Evaluation Measures (%)					
	TPR	TNR	PPV	F-Score	AUC	Accuracy
1	87.75	99.20	99.67	93.33	93.47	90.75
2	99.16	90.75	96.97	97.74	94.95	97.05
3	91.64	100	100	95.63	95.82	94.10
4	94.85	99.20	99.67	97.20	97.02	95.90
5	99.72	93.38	97.78	98.74	96.55	98.10
Mean ± SD	94.62±2.27	96.5±1.86	98.18±0.61	96.53±0.94	95.56±0.63	95.18±1.3

**Table 8:** Performance measures of the proposed CNN architecture: Nadam optimization.

5-FCV (Fold wise)	Evaluation Measures (%)					
	TPR	TNR	PPV	F-Score	AUC	Accuracy
1	99.42	100	100	99.71	99.71	99.58
2	100	98.47	99.42	99.71	99.23	99.58
3	99.44	99.14	99.72	99.57	99.29	99.36
4	99.16	99.16	99.71	99.43	99.16	99.16
5	100	99.24	99.71	99.85	99.62	99.78
Mean ± SD	**99.60±0.17**	**99.2±0.24**	**99.71±0.09**	**99.65±0.07**	**99.4±0.11**	**99.5±0.106**

**Table 9:** Performance measures of the proposed CNN architecture: RMSProp optimization.

5-FCV (Fold wise)	Evaluation Measures (%)					
	TPR	TNR	PPV	F-Score	AUC	Accuracy
1	99.72	88.13	96.22	97.94	93.92	96.85
2	100	73.68	90.71	95.13	86.84	92.63
3	94.67	99.15	99.70	97.12	96.91	95.78
4	99.41	97.74	99.12	99.26	98.57	98.94
5	99.13	93.22	95.03	97.04	96.17	95.57
Mean ± SD	98.58±0.98	90.38±4.6	96.15±1.61	97.3±0.67	94.48±2.05	95.95±1.02

**Note**: *SD stands for standard deviation

### 4.2. Segmentation of abnormal brain MR images

**Fig 7:**
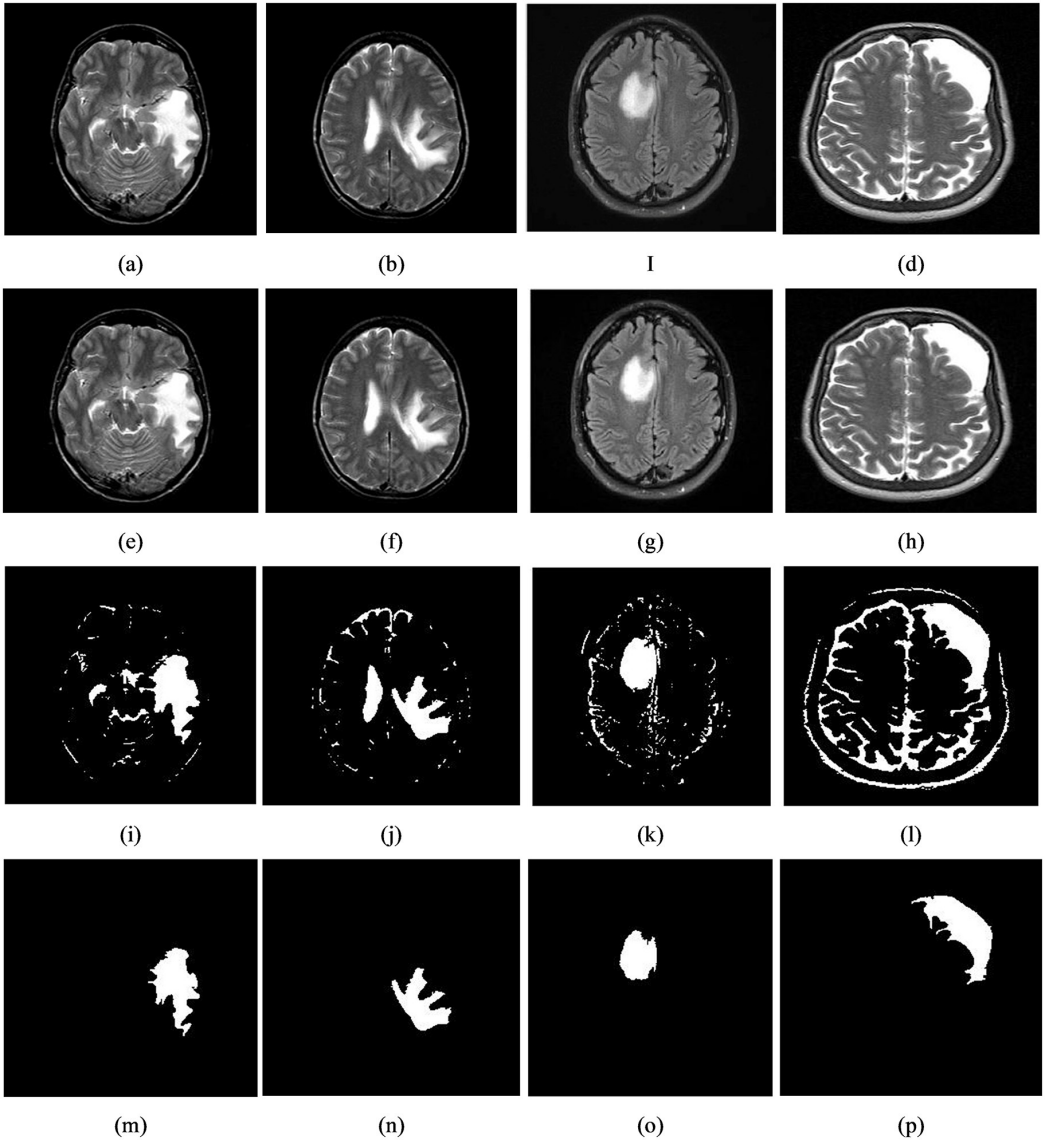
The results of the applied segmentation technique to detect single tumor can be observed in four sets of images. **(a)-(d)** shows the original input images, **(e)-(h)**, the images have undergone contrast enhancement through intensity transformation to improve the visibility of the tumors, **(i)-(l)**, the segmentation technique has been applied using multi-level thresholding to distinguish the tumor regions from healthy, **(m)-(p)**, refined segmented images.

**Fig 8:**
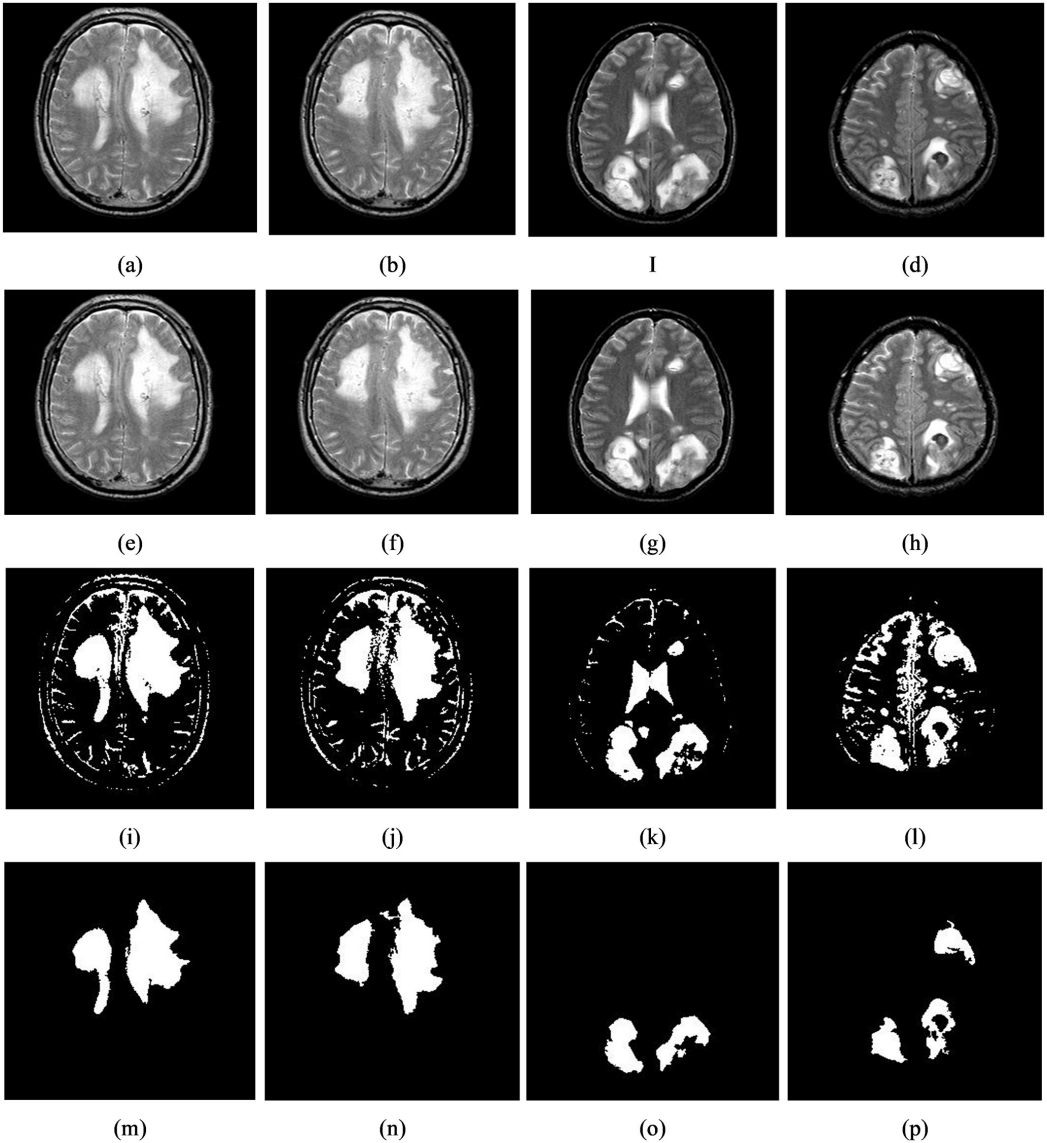
The results of the applied segmentation technique to detect multiple tumors can be observed in four sets of images. (a)-(d) shows the original input images, (e)-(h), the images have undergone contrast enhancement through intensity transformation to improve the visibility of the tumors, (i)-(l), the segmentation technique has been applied using multi-level thresholding to distinguish the tumor regions from healthy, (m)-(p), refined segmented images.

Brain tumor segmentation from MR images is a method for differentiating the diseased tissue from the healthy tissue to differentiate the infected area from its non-infected area at the pixel level. To meet this criterion, we proposed Tsallis entropy and DE-based multi-level thresholding approach in this work. Here, initially, partitions the image into six classes (e.g., class 1, class 2… class 6) using randomly initialized thresholding values says that t_1_, t_2_… t6 (t_1_ < t2 < t_3_ <…. < t_6_). Afterward, we calculate the Tsallis entropy from each class and then estimate the optimal thresholding value with the help of DE. The implications of the suggested segmentation model on single and multiple tumors are illustrated in Figs 7 and 8. Further, to test the impact of the proposed segmentation, we collected 25 infected brain tumor images from the database mentioned in section 3.1.1 and the corresponding inferences are shown in Table 10 and the highest attained measures are highlighted in bold face.

**Table 10:** Evaluation of metrics of the suggested segmentation strategy.

Images	DSC	PPV	TPR	TNR	F-Score	AUC	Accuracy
1	90.25	99.94	99.14	98.64	99.53	98.89	99.12
2	90.28	99.94	99.65	96.84	99.79	98.25	99.6
3	92.57	99.48	99.3	93.63	99.38	96.46	98.87
4	92.05	99.6	99.2	94.53	99.39	96.87	98.88
5	97.36	99.8	99.95	95.83	99.87	97.89	99.76
6	95.78	99.91	99.91	95.78	99.91	97.85	99.83
7	88.62	99.96	99.06	99.09	99.51	99.08	99.06
8	96.86	99.47	99.77	99.24	99.87	99.51	99.75
9	94.02	99.93	99.67	97.87	99.8	98.77	99.62
10	96.71	99.99	99.91	99.16	99.95	99.53	99.9
11	95.34	99.4	99.17	96.08	99.28	97.62	98.76
12	95.59	99.4	99.68	94.31	99.53	96.99	99.16
13	91.28	99.55	99.28	93.16	99.41	96.22	98.9
14	89.94	100	98.73	100	99.36	99.37	98.8
15	89	99.99	98.06	99.94	99.02	99	98.2
16	88.6	100	99.82	100	99.91	99.91	99.83
17	91.58	98.93	99.56	88.44	99.24	94	98.61
18	89.97	99.36	99.31	90.32	99.34	94.82	98.76
19	95.48	99.95	99.85	97.76	99.9	98.81	99.81
20	90.47	100	99.47	99.82	99.73	99.64	99.48
21	94.94	100	99.45	100	99.72	99.73	99.48
22	96.46	99.94	99.85	97.91	99.89	98.88	99.79
23	91.58	99.78	99.48	94.79	99.63	97.14	99.29
24	91.08	100	99.47	100	99.73	99.73	99.48
25	95.32	99.77	100	91.42	99.88	95.71	99.77
**Average**	**92.84**	**99.76**	**99.47**	**96.58**	**99.62**	**98.03**	**99.3**

## 5. Discussion

Detecting brain tumors from MR images is a difficult and intricate task, mainly due to the impact of noise, limited data, and the movements of organs within the brain. To resolve these issues, researchers introduced various methodologies (refer to Section 2) based on fundamental steps involved in machine learning. However, they have a few problems (refer to Section 2.1). Hence, in this work, we presented a distinctive methodology for an early diagnosis of brain tumors using CNN and multi-level image thresholding. Table 11 indicates the classification performance of the suggested model with other state-of-the-art approaches, as discussed in Section 2. From this, we noted that the suggested diagnosis model attained approximately 1.2% higher accuracy than the conventional pre-trained CNN architectures [6, 9, 14, 20, 21, 23, 24] and other deep learning frameworks. This slight improvement is very important in the brain image analysis since brain tumor is life-threatening disease. The significant merits of the presented technique are:

1. Our model instantaneously reads the structure of brain MR images, extracts the hidden details for identifying abnormal patients, and minimizes the intervention of human beings.
2. Required a smaller number of parameters than the pre-trained CNN models, that is approximately 5, 53,794 (or 0.55 million), including 5, 49,890 trainable and 3904 non-trainable.
3. Overcome the over-fitting issues and improve the model generalization ability by introducing the weights into the convolutional layers using the concept of ‘He weight initialization’.
4. Significantly obtained high classification accuracy due to image augmentation.
5. Effectively extract the hidden texture details without the intervention of humans.

**Table 11:** Classification performance of the implemented approach and state-of-the-art works.

Methodology	Metrics (%)		
	TPR	TNR	Accuracy
BrainMRNet [8]	96	96.08	96.05
ANN [5]	90.9	96.78	94.07
AlexNet+VGG-16+RFE [6]	97.83	95.74	96.77
2D-CNN [12]	94.11	100	97.14
AlexNet [9]	100	75	95.71
DCNN [13]	100	96.67	98.28
NLCMFO + CNN [18]	96	98.6	97.4
EWS+ResNet 50 [19]	96.67	84.21	92
VGG-19 [20]	100	94.73	98
VGG-16+SVM [14]	93.04	82.16	88.35
GLCM+ANFIS [7]	96.2	97.7	98.7
CS+KNN [16]	90.3	91	98.12
ELBP+SVM [11]	98.48	94.28	97.02
PDCNN [25]	95.65	100	97.33
Deep CNN [23]	96.88	96.88	96.88
CNN [24]	98.20	98.33	98.20
MHO-CNN [27]	98.7	98.3	98
SVM [28]	94.2	84.78	90.68
Hybrid Approach [29]	100	90.9	96.7
VGG 19+SSA+Cubic SVM [30]	99	98.36	99.1
**The Proposed Model**	**99.6**	**99.2**	**99.5**

Similarly, when comparing our method with the existing segmentation models, we found that a 3% increase in DSC value (Table 12). In the medical imaging, this improvement is crucial because it improves the efficiency of diagnostic tool. The main motives behind the success of the suggested segmentation model are as follows:

Tsallis entropy is ability to analyze the non-extensive details.

1. Tsallis entropy is taken into consideration of correlation between sub-samples due to its pseudo-additivity property.
2. Differential evolution requires less parameter tuning, fast and accurate convergence towards finding a global minimum.

**Table 12:** Segmentation performance of the implemented model and state-of-the-art works.

Methodology	DSC
NLR-MRF [10]	0.77
SOM +FKM [4]	0.47
KFCM +PSO [15]	0.85
Cuckoo Search [16]	0.9
FCM +PSO [22]	0.87
IMV-FCM [17]	0.89
CNN+RPCA [26]	0.91
**The Proposed Model**	**0.93**

### 5.1. Advantages

Major advantages of the proposed model compared to the existing approaches stated above are as follows:

- **Efficiency:** Lightweight CNN architectures require less training time and computational resources compared to complex models. This makes them suitable for deployment on devices with limited processing power.
- **Improved Classification Accuracy:** The use of a CNN can potentially achieve higher accuracy in classifying normal and abnormal MR images compared to traditional machine learning methods. This leads to a more reliable identification of potential tumor cases.
- **Better Tumor Segmentation:** The Tsallis entropy and DE-based multi-level thresholding approach offers a clear method for segmenting the tumor region at the pixel level. This provides a detailed map of the diseased tissue, aiding in treatment planning and surgical procedures.
- **Potential for Automation:** By automating the classification and segmentation process, this methodology can significantly reduce the workload for radiologists and improve the efficiency of brain tumor diagnosis.

### 5.2. Limitations

As we know every approach have some pros and cons, here, we listed a few limitations of the presented framework:

- **Sensitivity to Training Data:** Lightweight CNN’s can be sensitive to the quality and size of the training dataset. Limited data or data with specific biases might affect the model’s generalization.
- **Requirement for Pre-processing:** The Tsallis entropy and DE-based approach might require specific pre-processing steps for optimal performance. This can add complexity to the overall pipeline.
- **Potential for Inaccuracy:** Thresholding techniques can be susceptible to noise and artifacts in MR images, leading to inaccurate segmentation boundaries.

### 5.3. Future scope

To limit the above-mentioned issues, in the future our work is extended using the following technologies:

- **Deep Learning Integration:** Explore integrating the Tsallis entropy and DE-based approach with deep learning architectures for potentially improved segmentation accuracy and robustness to noise.
- **Real-time Applications:** Investigate methods to optimize the methodology for real-time applications, such as image-guided surgery or intra-operative tumor identification.
- **Explainable AI:** Explore techniques for making the CNN model more interpretable. This can help healthcare professionals understand the rationale behind the model’s predictions and build trust in its results.
- **Generalizability Studies:** Conduct studies to evaluate the generalizability of the methodology on diverse datasets with different types of brain tumors and image acquisition protocols.
- **Incorporation of Clinical Data:** Consider incorporating additional clinical data (e.g., patient history) into the model to potentially improve the accuracy of both classification and segmentation.

## 6. Conclusion

In this study, we developed a new methodology to distinguish between normal and abnormal MR images by identifying the infected areas from brain tumor images. Here, preprocessing is primarily used to reduce the effects of unwanted artifacts that happen while capturing MR images. Then, we used image augmentation based on geometric transformations to improve the predictive model’s performance. Further, we extracted the hidden texture details from the augmented images and classified them as normal and abnormal by the suggested CNN architecture. Finally, MR images of the pathological brain were subjected to multi-level thresholding to isolate the region of interest. From the empirical findings of brain MR images, we witnessed that our suggested model identifies abnormal patients with an accuracy of 99.5%. From the detailed analysis of experimental assessments, it is noted that compared to the existing approaches, the proposed methodology accurately classifies the given brain MR images as normal and abnormal with 99.5% accuracy and effectively identifies the location of affected regions with 0.93 DSC. Hence, the presented technique can be used as a powerful tool for MR-based brain tumor classification and identification. In the future, we would like to extend our work on 3-dimensional brain MR images.
